# Supplementary material for: WordCommentsAnalyzer: A windows software tool for qualitative research
Source: F1000Res. 2018 Sep 4;7:536. Originally published 2018 May 3. [Version 2] doi: 10.12688/f1000research.14819.2 (PMC6137411; doi:10.12688/f1000research.14819.2)
Supplement: Supplementary file 3 [file f1000research-7-17614-s0002.tgz › b2a79496-4ada-47c4-9682-a99ce867836d.zip!/Eight Iranian Health Journals/Yafteh _42.docx]

© Yafteh  . This work is licensed under CC BY 4.0 (https://creativecommons.org/licenses/by/4.0/).

Volume & Issue: دوره 10 - شماره 3

Title: بررسی مقایسه ای تاثیر مصرف داروهای اولانزاپین و ریسپریدون بر سطح چربی و قند خون و سایر عوارض در اختلالات سایکوتیک

http://yafte.lums.ac.ir/article-1-84-fa.html

مقدمه: بیماریهای مزمن از معضلات جدی موجود در روانپزشکی می باشند. داروهای آنتی سایکوتیک آتیپیک از داروهـای جدیدموثردردرمان این اختلالات به حساب می آیند، متاسفانه این داروها با بروز عوارضی نظیر افزایش قند و چربی خون، افزایش وزن و ادم جنرالیزه همراه می شوند. پــژوهش حاضر با هـدف بررسـی مقایسه ای تاثیر مصـرف دو داروی اولانزاپین و ریسپریدون بر سطـح چربی و قـند خون و سایر عوارض در بیماران مبتلا به اختلالات سایکوتیک انجـام گرفته است. مواد و روش‌ها: مطالعه حاضر یک کار آزمایی بالینی دو سوکور تصادفی است که در آن بـیـماران مبتلا بـه اخـتـلالات سایکو تیک بطور تصادفی در دو گروه تحت درمان با اولانزاپین و ریسپریدون قرارگرفتند. کلیه نمونه ها در ابتدای مطالعه از نظر قند خون و لیپیدها مورد ارزیابی قرار گرفتند و در صورت نرمال بودن آزمایشات، نمونه ها بطورتصادفی دوسوکور در یکی از گروههای تحت درمان با اولانزاپین یا تحت درمان با ریسپریدون قرار گرفتند. آزمایشات قند خون و لیپیدها در هفته اول و ماه سوم بعد از شروع درمان جهت کلیه نمونه ها انجام و از نظر سایر عوارض مورد ارزیابی قرار گرفتند. اطلاعات پس از جمع آوری از طریق نرم افزار spss مورد تجزیه و تحلیل قرار گرفت. Ø یافته‌ها: یافته های تحقیق نشان داد که سطح کلسترول و تری گلیسرید و قند خون بیماران در هفته اول و ماه سوم بعد از شروع درمان بطور معنی داری افزایش یافته بود که این افزایش سطح کلسترول و تری گلیسرید در دو گروه اولانزاپین و ریسپریدون تفاوت معنی داری داشت در حالیکه میزان قند خون در این دو گروه تفاوت معنی داری نداشت. وجود عوارض جانبی نظیر: بیقراری، ناتوانی جنسی، افزایش وزن، ادم و خواب آلودگی در دو گروه اختلاف معنی داری داشت. بحث و نتیجه‌گیری: با توجه به نتایج روشن این تحقیق بنظر می رسد قبل از تجویز داروهای آنتی سایکوتیک آتیپیک، بررسی آزمایشات چربی و قندخون در این بیماران ضروری است در حالیکه در هیچیک از منابع اصلی آموزشی روان پزشکی این گونه عوارض، هیپرتری گلیسریدمی،هیپر کلسترومی و هیپرگلیسمی و سایر عوارض جدی تلقی نشده است. توصیه می گردد در بیمارانی که دچار مشکلاتی نظیر افزایش لیپیدها و یا اختلال در تحمل گلوکز می باشند مصرف دارو با احتیاط بیشتر و پیگیری منظم مقادیر آزمایشگاهی حیاتی به نظر می انجام می گیرد.

Title: اندازه گیری‌ فعالیت‌ آنزیم‌ سیالیل‌ ترانسفراز و اثرات‌ عمل فسفریلاسیون‌ و دفسفریلاسیون‌ بر فعالیت‌ آن ‌در مغز موش‌آزمایشگاهی

http://yafte.lums.ac.ir/article-1-85-fa.html

مقدمه: گزارش ‌های‌ قبلی‌ نشان‌ می‌دهد که‌ مکانیزم‌ فسفریلاسیون‌ و دفسفریلاسیون‌ در تنظیم ‌فعالیت‌ آنزیم‌ سیالیل‌ ترانسفراز دخالت‌ دارند. در این‌ پژوهش هدف بررسی تغییرات فعالیت ‌آنزیم‌ سیالیل‌ ترانسفراز از طریق‌ مکانیسم‌ فسفریلاسیون‌ و دفسفریلاسیون است. مواد و روش‌ها: پژوهش حاضر بر روی‌ 25 موش‌ آزمایشگاهی ‌انجام‌ شد. مغز موشها را بیرون آورده ‌و هموژنه‌ مغز تهیه‌ شد. جدا سازی آنزیم سیالیل ترانسفراز مغز موش‌آزمایشگاهی‌ در کروماتوگرافی‌ ستونی‌ سفادکس‌ 50 G - انجام‌ شد. فعالیت آنزیم در حضور پروتئین‌ کیناز c ، فس ف اتاز، اکادئیک‌ اسید و فوربل‌ ‌اندازه گیری شد. نتایج‌ توسط‌ نرم‌افزار آماری‌ SPSS آنالیز شد . یافته‌ها: مقایسه‌ فعالیت‌ سیالیل‌ ترانسفراز در حضور پروتئین‌کیناز c واکادئیک‌ اسید با گروه‌ کنترل‌ نشان‌ داد که‌ سطح‌ فعالیت‌ سیالیل‌ ترانسفراز نسبت به گروه‌کنترل‌ پایین تر و از نظر آماری‌ معنی دار‌ بود (05/0 p< ) . مقایسه‌ فعالیت‌ سیالیل‌ ترانسفراز در حضور پروتئین ‌فسفاتاز و فوربل‌ نشان ‌داد که‌ در گروه‌ کنترل‌ پایین‌تر و نسبت به حضور پروتئین کیناز بالاتر بود و از نظر آماری معنی دار‌ بود . بحث و نتیجه‌گیری: یافته‌های‌ پژوهش حاضر نشان‌ داد که‌ واکنش‌ سیالیل‌ ترانسفراز مغز موش ‌آزمایشگاهی‌ با پروتئین‌کیناز c فعالیت‌ آنزیم‌ را کاهش‌ می‌دهد و این‌ نتایج‌ با یافته‌های‌ سایر محققان‌ منطبق‌ است‌. ما در یافتیم‌ که‌ استفاده‌ از سیالیل‌ ترانسفراز همراه‌ با پروتئین‌ کیناز c باعث‌ کاهش‌فعالیت‌ آن‌ می‌شود و پروتئین فسفاتاز فعالیت آن را افزایش می دهد. در نهایت فسفریلاسیون سبب کاهش فعلیت آنزیم سیالیل ترانسفراز و دفسفرسیلاسیون سبب افزایش فعالیت آن می گردد .

Title: بررسی میزان فراوانی دلیریوم در بیماران بالای 18 سال بستری در بخش های داخلی و جراحی بیمارستان شهدای عشایر خرم آباد

http://yafte.lums.ac.ir/article-1-86-fa.html

مقدمه : دلیریوم در افراد مسن و بیماران بستری خصوصاً پس از اعمال جراحی به وفور دیده می‌شود، لیکن درصد بالایی از موارد بدون تشخیص و درمان مانده و بیماران دچار پیامدهایی نظیر افزایش مرگ و میر، عوارض، کاهش بقاء، افزایش مدت بستری در بیمارستان و عوارض ناتوان کننده‌ی ذهنی بعدی می‌گردند. متاسفانه علیرغم اهمیت این سندرم، پزشکان و پرسنل تنها قادر به تشخیص کمتر از یک سوم بیماران می‌باشند. مواد و روش‌ها: در این مطالعه مقطعی تعداد 240 نفر بیمار بستری به صورت تصادفی از بخش های داخلی و جراحی (هر یک 120 بیمار) بیمارستان شهدای عشایر خرم آباد انتخاب شده بود. ملاک تشخیصی دلیریوم آزمون بررسی کننده ی وضعیت شناختی فرد یا MMSE بود وهر بیمار چهار روز متوالی مورد بررسی قرارگرفت. یافته‌ها: در بخش داخلی از 120 بیمار، 37 مورد (8/30%) و در بخش جراحی نیز از 120 بیمار، 25 مورد(8/20%) ابتلا به دلیریوم وجود داشت. در بخش داخلی 27 مورد (5/22%)و در بخش جراحی نیز 27 مورد (5/22%) مشکوک به دلیریوم وجود داشت. در بخش جراحی بیشترین فراوانی دایریوم در گروه های سنی 77-58 سال و بالای 77 سال و در بخش داخلی بیشترین فراوانی دلیریوم در گروه های سنی بالای 77 سال بود. در بخش جراحی از نظر ابتلا به دیلیریوم بین دو جنس ارتباط معنی داری وجود داشت(1 0/0 p= ). ولی در بخش داخلی از نظر ابتلا به دلیریوم بین دو جنس تفاوت معنی داری جود نداشت( 92 /0 p= ). بحث و نتیجه‌گیری: با توجه به فراوانی سندرم دلیریوم در بخشهای مختلف داخلی و جراحی جامعه ما، وجود روان‌پزشک مشاورـ در جهت تشخیص زود رس و کنترل دلیریوم در بیمارستان های داخلی و جراحی ضروری به نظر می رسد .

Title: بررسی آلودگی باکتریال کیست های هیداتیک حیوانی و تعیین نوع باکتری های مولد عفونت در حیوانات ذبح شده در کشتارگاههای همدان و بروجرد سال 1385

http://yafte.lums.ac.ir/article-1-87-fa.html

مقدمه: کیست هیداتیک مرحله لاروی انگل اکینوکوکوس گرانولوزوس است که در امعاء و احشاء حیوانات و انسان ایجاد می گردد. گاهی نفوذ باکتری ها بدرون مایع کیست به دلایل نامعلوم، منجر به عفونی شدن آنها و مانع از تولید لارو یا پروتواسکولکس می شود و کیست عقیم می گردد. در این مطالعه میزان آلودگی باکتریال و انواع باکتری های مولد عفونت در کیست های هیداتیک دامی در کشتارگاه های همدان و بروجرد مورد مطالعه و مقایسه قرار گرفته است. مواد و روش‌ها: با مراجعه به کشتارگاه های همدان و بروجرد تعداد 5709 راس دام از نظر وجود کیست بررسی شدند. در دامهای آلوده، عضو مبتلا، تعداد کیست، اندازه آن، باروری یا عقیم بودن آن، وجود آلودگی باکتریال در کیست و.... مشخص شد. مقداری از مایع هیداتیک در محیط های مناسب کشت باکتری کشت داده شد و باکتری های ایزوله شده با روش های بیوشمیایی تعیین هویت گردیدند. یافته‌ها: میزان آلودگی به کیست هیداتیک در دامهای همدان 5/6% و در دامهای بروجرد 7% بود. در دامهای همدان تنها 20% کیست ها بارور و در دامهای بروجرد 32% کیست ها بارور بودند. در همدان 74% و در بروجرد 62% کیست ها عفونی بودند. باکتری های جدا شده از کیست های عفونی همدان و بروجرد تقریبا مشابه و شامل اشرشیا کلی، کلبسیلا، پروتئوس، انتروباکتر، استافیلوکوک طلائی و استافیلوکوک های غیر همولیتیک، سودوموناس و ادواردسیلا بود. در همدان 81% باکتری ها مولد توکسین (از نوع اگزوتوکسین و یا اندوتوکسین) بودند و در 19% بدلیل اینکه گونه آنها تشخیص داده نشد در مورد توکسینوژن بودن آنها اظهار نظر نگردید و در بروجرد ارقام فوق به ترتیب 93% و 7% بود. بحث و نتیجه‌گیری: این مطالعه نشان داد که میزان آلودگی دامهای کشتاری به کیست هیداتیک در همدان و بروجرد تفاوت معنی داری با هم ندارد. درصد بسیار بالایی از کیست ها در هر دو شهرستان آلوده به باکتری هستند که نوع غالب باکتری ایجاد کننده عفونت در کیست ها در هر دو شهرستان اشرشیا کلی و کلبسیلا می باشد.

Title: تاثیر زایمان در آب بر طول مدت زایمان و میزان درد در مقایسه با زایمان به روش متداول

http://yafte.lums.ac.ir/article-1-88-fa.html

مقدمه: با توجه به هدف سازمان بهداشت جهانی و وزارت بهداشت و درمان مبنی بر کاهش میزان سزارین باید به دنبال روشهای جایگزین سزارین انتخابی باشیم. به همین دلیل برآن شدیم اثرات زایمان در آب را به عنوان یک روش زایمان طبیعی بدون مداخله پزشکی بر کاهش میزان درد و طول مدت زایمان مورد ارزیابی قرار دهیم. مواد و روش‌ها: این مطالعه از نوع کارآزمایی بالینی بوده و جامعه مورد پژوهش خانمهای باردار G1 و G2 با سن حاملگی 38-42W بوده اند که به درمانگاه بیمارستان عسلیان مراجعه کرده بودند. حجم نمونه 100 مورد بود که در دو گروه 50 نفری زایمان به روش معمولی و زایمان در آب قرار گرفتند. و طول مدت زایمان و میزان درد با استفاده از تستهای آماری کای اسکور، کروسکال والیس و من وینتنی مورد تجزیه و تحلیل آماری قرار گرفت و 05/0 p< معنی دار تلقی گردید. یافته‌ها: نتایج نشان داد که میانگین طول مدت مرحله فعال زایمان در روش زایمان در آب 8/0 ± 1/3 ساعت بود که در مقابل طول مدت مرحله فعال زایمان در گروه مقابل که 8/0 ± 7/4 ساعت بوده به طور معنی داری کاهش دارد (05/0 p< ) و همچنین طول مدت مرحله دوم زایمان در گروه زایمان در آب 22/0 ± 53/0 ساعت و در گروه مقابل 43/0 ± 88/0 ساعت بود که باتوجه به 05/0 p< به طور معنی داری طول مدت مرحله دوم زایمان نیز در روش زایمان در آب کاهش داشته است.میزان درد نیز در هر دو گروه با استفاده از روش میزان معیار درد تعیین شد. در گروه زایمان در آب 79/0 ± 53/3 و در گروه مقابل 7/1 ± 96/6 برآورد شد. که با توجه به 05/0 p< کاهش میزان درد در گروه زایمان در آب معنی دار تلقی گردید. بحث و نتیجه‌گیری: این مطالعه نشان می دهد که زایمان در آب به علت کاهش طول مدت زایمان و کاهش میزان درد و کاهش نیاز به مداخلات پزشکی می تواند جایگزین مناسب غیر دارویی و غیر تهاجمی برای موارد سزارین های انتخابی باشد.

Title: تاثیر استرس مزمن در مادران باردار بر پاسخگویی به مورفین در موشهای کوچک آزمایشگاهی به روش حساسیت رفتاری

http://yafte.lums.ac.ir/article-1-89-fa.html

مقدمه: مکانیسم هایی که زمینه ساز تمایل افراد به مصرف مورفین هستند هنوز به درستی شناخته نشده اند. طی تحقیقات گذشته مشخص شده است که تمایل افراد به مصرف مواد مخدر در هنگام استرس یا بعد از آن افزایش می یابد. در این تحقیق ما با استفاده از استرس بی حرکتی که نوعی از استرس روانی می باشد، تغییر تمایل نسل دوم را به مورفین با استفاده از روش القاء حساسیت رفتاری مورد بررسی قرار دادیم . مواد و روش‌ها: این پژوهش از نوع تجربی مداخله گر بود.که ابتدا موشهای کوچک آزمایشگاهی نر و ماده با هم جفت شدند. پس از حصول اطمینان از باردار بودن موش های ماده، به آنان توسط دستگاه مخصوصی تا زمان زایمان استرس داده شد. سر تعدادی از جنین ها جهت بررسی های بافت شناسی برش داده شد.پس از رسیدن بقیه جنین ها به سن بلوغ،حیوانات به منظور مشخص نمودن ترجیح چپ و راست توسط ماز – تی شکل ( (T-Maze مورد بررسی قرار گرفته و با گروه کنترل مقایسه شدند. به منظور تعیین اثر بخشی تجویز حاد مورفین،5 گروه حیوان استرس دیده و 5 گروه حیوان گروه استرس ندیده، انتخاب شدند. حیوانات هر دو گروه به گروههای کنترل (بدون تزریق)، سالین، مورفین mg/kg 1، مورفین mg/kg 10 و مورفین mg/kg 50 تقسیم شدند. که پس از تزریق توسط دستگاه (Open Field) مورد آزمایش حرکت سنجی قرار گرفتند.گروههای گفته شده در آزمایش قبلی 48 ساعت پس از تجربه اول به وسیله ی تجویز مورفین mg/kg 1 به منظور تعیین حساسیت القاء شده توسط تجویز قبلی مورفین، مجدداً مورد آزمایش حرکت سنجی قرار گرفتند . یافته‌ها: یافته ها نشان داد که ضخامت قشر ناحیه فرونتال درگروه استرس دیده بسیار کمتر از گروه کنترل بود(01/0 p< ). هم چنین میزان راست برتری در موشهای نر گروهی که مادرانشان استرس دیده اند بسیار کم وراست برتری در موش های ماده ی این گروه زیاد بود (001/0 P< ) در مورد موش های نر و (01/0 P< ) در مورد موش های ماده، تجویز مورفین در گروه آزمایش در دوز کم بی اثر و در دوز زیاد باعث القا فعالیت حرکتی گردید اما این فعالیت حرکتی از نظر مقدار بسیار کمتر از گروه کنترل موجود بود. تجویز دوز کم مورفین mg/kg) 1) در گروههای استرس دیده باعث القا فعالیت حرکتی شد که این فعالیت در دوز mg/kg 50 آن از نظر آماری معنی دار بود. بحث و نتیجه‌گیری: نتایج نشان داد که القاء یک استرس روانی نسبتاً خفیف (بی حرکتی کوتاه مدت) توانست باعث بروز تغییرات عمده ای اولاً در مغز جنین، ثانیاً در ترجیح چپ و راست و ثالثاً در پاسخگویی به مورفین گردد.

Title: بررسی شیوع مشکلات رفتاری کودکان پیش دبستانی شهرستان خرم آباد 1385

http://yafte.lums.ac.ir/article-1-90-fa.html

مقدمه: دوران کودکی از مهمترین مراحل زندگی است که در آن شخصیت فرد پایه ریزی و شکل می گیرد. اغلب اختلالات رفتاری ناشی از کمبود توجه به دوران حساس کودکی است. این بی توجهی منجر به عدم سازش با محیط و بروز مشکلات رفتاری برای کودک می شود. مشکل رفتاری به رفتار فردی اطلاق می شود که بدون پایین بودن بهره هوشی، تعادل روانی و رفتاری از اندازه عمومی اجتماع دور و دارای شدت، تکرار، مداومت در زمانها و مکانهای متعدد باشد. به طوریکه در عملکرد تحصیلی، رفتاری دچار درماندگی و یا کاهش میزان کارایی فرد گردد، این گونه کودکان مرتب از سوی اطرافیان طرد میشوندو در مدرسه مرتب از انها گله و شکایت دارند. لذا توجه به مشکلات شایع رفتاری کودکان یکی از موضوعات حائز اهمیت وشناسایی سریع و به موقع اختلالات، امکان درمان سریعتر آنان را مهیا می ساز د. لذا مطالعه حاضر با هدف تعین شیوع مشکلات رفتاری در کودکان پیش دبستانی شهر خرم آباد 1385 انجام گرفت . مواد و روش‌ها: مطالعه فوق توصیفی تحلیلی مقطعی است که 600 کودک پیش دبستانی بر اساس شیوع 50% اختلالات رفتاری از شهر و روستا بروش تصادفی یک مرحله ای انتخاب شدند. ابزار گرد آوری داده ها پرسشنامه مشتمل دو بخش مشخصه های فردی و علایم رفتاری بر گرفته از DSM IV) ) در رابطه با علایم اختلالات رفتاری تنظیم شده بود. روایی صوری و محتوی فرم پرسشنامه توسط اساتید تایید و پایایی پرسشنامه با روش باز آزمایی با ضریب همبستگی 98% r= کسب شد اطلاعات در یک مرحله و با روش مصاحبه با مادر کودک در مدرسه توسط روانشناس کسب و سپس از نرم افزار spss 11 در قالب آمار توصیفی و استنباطی کای دو تجزیه وتحلیل شدند . یافته‌ها: نتایج نشان داد که3/79% از کودکان شهری و 68% کودکان روستایی حداقل گرفتار یکی از مشکلات رفتاری مورد مطالعه بودند. اضطراب جدایی، بهانه گیری زیاد و بی مورد، گریه فراوان، در کودکان شهری بالاتر و مشکلات رفتاری مثل دفع بی اختیار ادرار، وحشت شبانه، دندان قروچه، کوبیدن سر به در و دیوار، مشکلات خواب در کودکان روستایی بالاتر بود. برخی مشکلات رفتاری کودکان مانند دفع بی اختیار ادرار، لکنت زبان، جویدن ناخن و... با جنس رابطه معنی دار و بین سن، تحصیلات و شغل والدین، رتبه تولد و بعد خانوار با برخی مشکلات رفتاری ارتباط معنی داری حاصل گردید . بحث و نتیجه‌گیری: ای ن نتایج نشان داد که اکثر کودکان پیش دبستانی به نحوی از مشکلات رفتاری رنج می برند که نیازمند توجه و حمایت بیشتری در زمینه بهداشت روان هستند و این امر مستلزم یک برنامه ریزی جامع و دقیق در زمینه بهداشت روان می باشد. بدین منظور بررسی سطح سلامت روانی کودکان در پایگاه سنجش و ارائه خدمات بهداشت روان در مدارس و آموزش به والدین و مربیان به منظور پیشگیری از بروز اختلالات رفتاری و تشخیص و درمان به موقع آن در کودکان ضروری است .

Title: بررسی آلودگی بار میکروبی کل، کلی فرم و اشرشیا کلی شیرخام از مرحله دوشش تا تحویل به کارخانه در استان لرستان

http://yafte.lums.ac.ir/article-1-91-fa.html

مقدمه: کیفیت اولیه شیر خام متأثر از عواملی همچون تغذیه دام، سلا متی دام، ترکیب شیمیایی شیر و فعالیت میکروب موجود درآن بوده که بر روی محصولات نهایی تأثیرگذ ار می باشد. بنابر این کیفیت شیر خام به مدت زمان و شرایطی که شیر از تولید تا تحویل به کارخانه طی می کند بستگی تمام دارد. دراستان لرستان 97% شیر تولیدی گاوها از واحدهای کوچک و روستایی بد ست می آید. پژوهش فوق با هدف بررسی کانون های آلودگی شیرخام و شناخت نقاط بحرانی آلودگی شیر خام از مرحله دوشش تا تحویل به کارخانه در سال1382 انجام گرفت . مواد و روش‌ها: جهت نمونه گیری از شیرخام مراکز با ظرفیت جمع آوری شیر بیش از 3 تن، 5/1 تا 3 تن و کمتر از 5/1 تن به عنوان مراکز با ظرفیت بالا، متوسط و کم تعیین گردید. مراحل نمونه برداری شامل نمونه گیری از شیر پستان، ظروف جمع آوری و نگهداری شیر، مخرن ماشین سیار جمع آوری، مخزن سردکن مرکز جمع آوری و از تانکر حمل شیر به کارخانه بود. آزمایش شمارش کلی بار میکروبی( (TC وآزمایشهای شیمیایی شامل میزان اسیدیته و pH شیر بود. کل نمونه جمع آوری شده در هر ماه 45 نمونه و در طی 12 ماه 540 نمونه جمع آوری گردید. داده ها جهت تجزیه و تحلیل آمار به لگاریتم درپایه 10 تغییر یافته و توسط نرم افزار SPSS 11. 5 با استفاده از روش GLM ) ) آنالیزواریانس گردید . یافته‌ها: میانگین بار میکروبی (TC ) در کلیه فصول سال cfu/ml 37/0 ± 43/6 بود. اثر ماههای مختلف سال، مراحل مختلف نمونه گیری از شیر و ظرفیت مراکز جمع آوری بر روی بار میکروبی کل، کلی فرم و اشرشیا کلی کاملاً معنی دار بود (01/0 p< ) . نتایج نشان داد کمترین بار میکروبی در دی ماه با cfu/ml 38/0 ± 31/6 و بیشترین بار میکروبی در مرداد ماه بامیزان cfu/ml 31/0 ± 57/6 بود. نتایج نشان داد کاهش بار میکروبی از شهریور ماه شروع و در دی و بهمن به کمترین میزان می رسید. نتایج همچنین نشان داد بیشترین میزان باکتریهای کلی فرم و اشرشیا کلی در شهریور ماه بوده و کمترین میزان کلی فرم در شیر خام در دی و بهمن و اشرشیا کلی در بهمن و اسفند ماه بود. ضرایب همبستگی نشان داد بین بار میکروبی با کلی فرم (90/0 r= ) و با اشرشیا کلی (79/0 r= ) ، همبستگی مثبت و معنی داری وجود داشت (01/0 p< ) . بحث و نتیجه‌گیری: بطور کلی مشکل مهم جمع آوری شیر در استان لرستان، دوری برخی مراکز جمع آوری شیر و مدت زمان طولانی انتقال شیر از این مراکز تا کارخانه شیر بوده، که بعنوان یکی از علل مهم افزایش بار میکروبی کل از مرحله دوشش تا تحویل به کارخانه می باشد. علاوه بر این امکانات ارتباطی محدود برخی از روستاها باعث افزایش بار میکروبی شیر خام در این مناطق گردیده است .

Title: تعیین پتانسیل خورندگی یا رسوب گذاری آب آشامیدنی شبکه های توزیع شهر خرم آباد با استفاده از اندیس های خوردگی

http://yafte.lums.ac.ir/article-1-92-fa.html

مقدمه: بر اساس استاندارد آب آشامیدنی نباید خورنده باشد، آبهای خورنده مواد اولیه لوله ها، اتصالات و شیر آلات شبکه های توزیع شهری و خانگی را در خود حل کرده و مشکلات عدیده بهداشتی، زیباشناسی و اقتصادی را در سیستم های آبی به وجود می آورند و در صورت لزوم خورندگی باید با هدف سالم سازی آب آشامیدنی و حفظ و ارتقاء سلامت و بهداشت شهروندان کنترل گردد. بنا براین تعیین پتانسیل خوردگی آب آشامیدنی با روشهای قابل اعتماد و قابل اجرا ضروری است . مواد و روش‌ها: این تحقیق با هدف تعیین پتانسیل خورندگی یا رسوب گذاری آب آشامیدنی شبکه های توزیع شهر خرم آباد با استفاده از اندیس های خوردگی انجام گرفت. در این تحقیق، مقدار اندیس های لانژلیه، رایزنر، خوردگی و پوکوریوس با تعیین پارامترهای درجه حرارت، سختی کلسیم، قلیائیت، کل جامدات محلول و pH در 50 نقطه در شبکه توزیع در دو مرحله ودر فصول تابستان و پاییز محاسبه گردید . یافته‌ها: نتایج روش اندیس های خورندگی، شامل اندیس های لانژلیه (157/0-)، رایزنر (86/7)، خوردگی (626/11) و پوکوریوس (65/7) آب شهر را در شرایط متمایل به خورندگی نشان می دهد . بحث و نتیجه‌گیری: آزمون های آماری ضریب همبستگی و پیرسون و اسپرمن و t زوج بجز در چند مورد محدود، ارتباط معنی داری بین پارامترهای کیفی با اندیس های خوردگی را نشان می دهد. نتایج روش های خوردگی، اندیس لانژلیه، اندیس رایزنر، اندیس خورندگی و پوکوریوس آب آشامیدنی شهر را در شرایط متمایل به خورندگی نشان می دهد .

Title: بررسی میزان آلودگی ضایعات نان به آفلاتوکسین در استان لرستان

http://yafte.lums.ac.ir/article-1-93-fa.html

مقدمه: آفلاتوکسین‌ها،گروه بزرگی از مایکوتوکسین‌ها هستند که آلودگی به آنها می‌تواند موجب بروز عوارضی چون سندرم ریه، سرطان مری، تضعیف سیستم ایمنی و سرطان‌کبدی، جلوگیری از ساخته شدن RNA و پروتئین و هموآگلوتیناسیون در انسان می‌شوند. همچنین آلودگی با آفلاتوکسین عارضه‌های متعددی در دستگاه تناسلی، گوارشی و تنفسی ایجاد می‌کند و در اثر داشتن قدرت تومورزایی و مسمومیت، مایه‌ی بروز عوارض متعددی در دام‌ها می‌گردند. این تحقیق با هدف تعیین میزان و فراوانی آلودگی ضایعات نان به آفلاتوکسین در استان لرستان و مقایسه‌ آن با استانداردهای معتبر جهانی اعلام شده از سوی سازمان بهداشت جهانی( WHO ) و سازمان خواربار ملل متحد( (FAO ‌انجام گردید . مواد و روش‌ها: این تحقیق مقطعی به روش میدانی وآزمایشگاهی با نمونه‌گیری از180 نمونه ضایعات نان خشک درسال 1385و با توزیع تصادفی درسطح استان لرستان و تشخیص آلودگی نمونه‌ها به آفلاتوکسین با استفاده از دستگاه HPLC فلورسانس انجام گرفت. تجزیه و تحلیل داده‌ها با استفاده از نرم افزار SPSS و در سطح 5% صورت گرفت . یافته‌ها: میزان متوسط انواع آفلاتوکسین B1 ، B2 ، G1 ، G2 و مجموع انواع آفلاتوکسین در ضایعات نان خشک مورد بررسی(آلوده، نیمه آلوده و سالم) درسطح استان لرستان به ترتیب عبارت است از: 5304/22، 4369/2، 1923/0، 1022/0 و( µg/kg ) 2636/25. میزان متوسط کمترین و بیشترین آلودگی به آفلاتوکسین نوع B1 و مجموع انواع آفلاتوکسین به ترتیب مربوط به شهرهای خرم‌آباد ( µg/kg 9403/42 و µg/kg 7153/47)و بروجرد( µg/kg 8611/1 و µg/kg 9833/1)است.میزان متوسط آفلاتوکسین نوع B1 در انواع نان‌های آلوده، نیمه آلوده و سالم به ترتیب 0536/64، 9167/1 و( µg/kg ) 5629/0 و میزان متوسط مجموع انواع آفلاتوکسین در انواع نان‌های آلوده، نیمه آلوده وسالم به ترتیب 0257/72، 999/1 و( µg/kg )5753/0 است.همچنین میزان آفلاتوکسینِ نوع B1 29 نمونه از180 نمونه، بیش از حد استاندارد و میزان مجموع انواع آفلاتوکسینِ 18 نمونه نیز بیش از حد استاندارد بوده است. بحث و نتیجه‌گیری: نتایج مطالعات انجام شده نشان داد شیر گاوهایی که از نان خشک تغذیه می کنند دارای مقادیر فراوانی از متابولیت های آفلاتوکسین است بنابراین تا حد امکان باید از مصرف نان‌های خشک در تغذیه دام و مصرف انسانی خودداری کرد.
